# Elucidating the Supramolecular Interaction of Positively Supercharged Fluorescent Protein with Anionic Phthalocyanines

**DOI:** 10.1002/adbi.202400308

**Published:** 2024-10-16

**Authors:** Sharon Saarinen, Ramsha Khan, Marta Patrian, Juan Pablo Fuenzalida‐Werner, Rubén D. Costa, Petr Zimcik, Veronika Novakova, Tero‐Petri Ruoko, Nikolai V. Tkachenko, Eduardo Anaya‐Plaza, Mauri A. Kostiainen

**Affiliations:** ^1^ Department of Bioproducts and Biosystems Aalto University Aalto 00076 Finland; ^2^ Faculty of Engineering and Natural Sciences Tampere University Korkeakoulunkatu 8 Tampere 33720 Finland; ^3^ Technical University of Munich Campus Straubing for Biotechnology and Sustainability Chair of Biogenic Functional Materials Schulgasse, 22 94315 Straubing Germany; ^4^ Faculty of Pharmacy in Hradec Kralove Charles University Ak. Heyrovskeho 1203 Hradec Kralove 50003 Czech Republic

**Keywords:** artificial photosynthesis, biohybrid materials, energy transfer, phthalocyanines, positively supercharged fluorescent proteins, protein‐based materials

## Abstract

Developing bioinspired materials to convert sunlight into electricity efficiently is paramount for sustainable energy production. Fluorescent proteins are promising candidates as photoactive materials due to their high fluorescence quantum yield and absorption extinction coefficients in aqueous media. However, developing artificial bioinspired photosynthetic systems requires a detailed understanding of molecular interactions and energy transfer mechanisms in the required operating conditions. Here, the supramolecular self‐assembly and photophysical properties of fluorescent proteins complexed with organic dyes are investigated in aqueous media. Supercharged mGreenLantern protein, mutated to have a charge of +22, is complexed together with anionic zinc phthalocyanines having 4 or 16 carboxylate groups. The structural characterization reveals a strong electrostatic interaction between the moieties, accompanied by partial conformational distortion of the protein structure, yet without compromising the mGreenLantern chromophore integrity as suggested by the lack of emission features related to the neutral form of the chromophore. The self‐assembled biohybrid shows a total quenching of protein fluorescence, in favor of an energy transfer process from the protein to the phthalocyanine, as demonstrated by fluorescence lifetime and ultrafast transient absorption measurements. These results provide insight into the rich photophysics of fluorescent protein–dye complexes, anticipating their applicability as water‐based photoactive materials.

## Graphical abstract

Positively supercharged mGreenLatern protein is self‐assembled electrostatically with negatively charged zinc phthalocyanines to yield bio‐based photoactive materials in aqueous media. The addition of phthalocyanines results in the formation of large complexes fully quenching of the protein fluorescence. The results indicate an energy transfer from the protein to phthalocyanine.

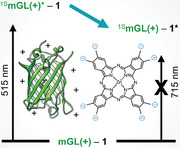

## 1 Introduction

Developing strategies to mimic photosynthesis within artificial systems with enhanced efficiency and robustness is an essential prerequisite for obtaining sustainable energy in the future.^[^ ^1^, ^2^, ^3^, ^4^ ^]^ Taking inspiration from nature, in which the noncovalent interactions between chlorophyll and proteins form the foundation of the light‐harvesting systems,^[^ ^5^ ^]^ supramolecular assembly holds the potential for building artificial light‐harvesting systems. In general, artificial light‐harvesting systems are often dissolved in organic solvents, given their highly hydrophobic nature, which leads to undesired aggregation‐caused quenching.^[^ ^6^ ^]^ There has been a drive to develop water‐processed light‐harvesting systems based on supramolecular self‐assembled systems^[^ ^7^, ^8^, ^9^ ^]^ or host‐guest chemistry, targeting applications such as water splitting.^[^ ^10^, ^11^ ^]^

Fluorescent proteins are promising water‐soluble photoactive materials, due to their high absorption extinction coefficients, high fluorescence quantum yields, and narrow emission in the visible spectrum. Furthermore, fluorescent proteins provide a tunable and sustainable solution for these applications as they are genetically encoded, straightforward to express in large quantities, and they can be recycled or disposed of via environmentally friendly processes.^[^ ^12^ ^]^ The potential of fluorescent proteins in applications such as Bio‐LEDs and lasers has been recently explored, revealing promising advancements in the field.^[^ ^13^, ^14^, ^15^, ^16^, ^17^, ^18^ ^]^ Furthermore, supercharged proteins offer an opportunity to use fluorescent proteins in photonic applications due to their ability to withstand thermally or chemically induced aggregation and denaturation.^[^ ^19^, ^20^, ^21^ ^]^ For instance, we have shown enhanced thermal stability and photoluminescence in electrostatic cocrystals of a supercharged variant of mGreenLantern (**mGL(+)**) and apoferritin proteins in Bio‐HLEDs.^[^ ^20^ ^]^ However, the use of fluorescent proteins as photosensitizers in artificial photosynthetic devices via supramolecular assembly has not been studied, and overall, their role in such devices is still relatively unexplored. Nevertheless, a few studies have investigated in this area. For example, the usage of red Kaede chromophore, derived from green fluorescent protein (GFP) chromophore, in organic‐based dye‐sensitized solar cells was demonstrated via functionalization of the synthesized chromophore onto a TiO_2_ nanocrystalline film.^[^ ^22^ ^]^ Moreover, a recent investigation provided an easy solution to implement fluorescent proteins in TiO_2_‐based dye‐sensitized solar cells decorating the surface of super folder green fluorescent protein (sfGFP) and mCherry with alkoxysilane groups that enable a straightforward device integration with stabilities over months under device working conditions.^[^ ^23^ ^]^ Furthermore, the incorporation of red‐emitting mScarlet fluorescent proteins in luminescent solar cell concentrators in a liquid‐state has been shown to retain the fluorescent quantum yield in comparison to solid‐state devices.^[^ ^24^ ^]^ Finally, GFP variants have also shown to be promising candidates in biophotovoltaic nanodevices.^[^ ^25^ ^]^

Among the plethora of artificial photosensitizers, phthalocyanines (Pcs) hold a prominent position among the porphyrinoid family. They have high thermal and chemical stability and show intense Q‐band absorbance in the red/near‐infrared region.^[^ ^26^, ^27^ ^]^ Besides, the photophysical properties can be tailored with macrocycle substitution.^[^ ^27^, ^28^ ^]^ However, due to the hydrophobic nature of the highly aromatic core, Pcs tend to aggregate in aqueous media, thus decreasing or entirely quenching their photophysical activity. Several chemical strategies have been employed to prevent the aggregation, including axial modification,^[^ ^29^, ^30^, ^31^ ^]^ electrostatic repulsion by decorating their peripheral positions^[^ ^32^ ^]^ with cationic^[^ ^33^, ^34^, ^35^ ^]^ or anionic^[^ ^36^, ^37^, ^38^ ^]^ substituents, or via supramolecular assembly together with, e.g., biomolecules^[^ ^39^, ^40^, ^41^, ^42^, ^43^, ^44^, ^45^ ^]^ for increased stability. Monomerically dissolved Pcs in aqueous media have shown their applicability as reactive oxygen species generators, and thus have applications as medicinal photosensitizers in photodynamic therapy.^[^ ^46^, ^47^, ^48^ ^]^ In addition, Pcs have been explored as light‐harvesting components in artificial photosynthetic systems, either self‐assembled or in the presence of other photoactive molecules.^[^ ^9^, ^49^, ^50^ ^]^

Herein, we present a photoactive bioinspired material via electrostatic self‐assembly. To this end, we study the structural and photophysical properties of oppositely charged fluorescent protein–organic dye complexes. The above‐mentioned, supercharged mGreenLantern protein variant was complexed with hydrophilic anionic ZnPcs to yield optically active complexes with rich photophysical properties. We report the structural characteristics of this complex along with its photophysical properties that support its potential as a photoactive material.

## 2 Results and Discussion

**Figure 1:**
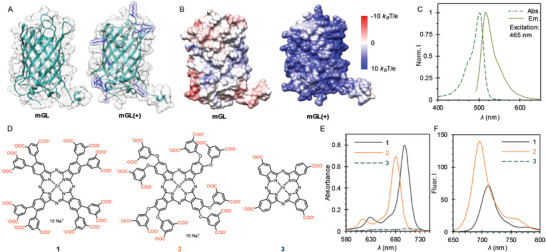
Structure of the studied fluorescent proteins and Pcs. A) The structures of native mGreenLantern (**mGL**, left) and supercharged (**mGL(+),** right) proteins. The mutated amino acid residues are shown in blue. B) Electrostatic potential visualization of the **mGL** (left) and **mGL(+)** (right). C) Absorption and emission spectra of **mGL(+)** (λ_ex_ = 465 nm). D) Molecular structures of the ZnPcs. The carboxylate groups are highlighted in red. E) Absorption and F) emission spectra of the ZnPcs at 10 µm concentration in 20 mm Tris, pH 8 (*λ*_ex_ = 635 nm for **1** and **3**, and *λ*_ex_ = 620 nm for **2**).

Complexation between phthalocyanines and fluorescent proteins was studied by characterizing the structural and photophysical properties of the complexes. Positively supercharged mGreenLantern protein, **mGL(+)**, with a charge of +22 was used. In addition, its native form (**mGL**), with a total surface charge of −2, was utilized as a control (**Figure** **1**A, right and left, respectively). The supercharged protein was designed by mutating 11 peripheral amino acids of the native protein without significantly affecting the conformational stability.^[^ ^20^ ^]^ The calculated electrostatic surface potential in aqueous media at pH 8 shows an increased positive charge for **mGL(+)** compared to the native form (Figure 1B). The **mGL(+)** exhibits an absorption band within the visible region, peaking at 502 nm, whereas the fluorescence maximum is observed at 518 nm (Figure 1C).

On the other hand, three negatively charged ZnPc (Figure 1D) were used as synthetic counterparts for the proteins. Compounds **1** and **2** (used in the form of their sodium salts) contain sixteen carboxylate moieties in their peripheral positions, which results in hexadeca‐anionic derivatives at physiologically relevant pH. In compound **1**, the isophthalyl fragment, acting as the anionic peripheral substituent, is directly attached to the phthalocyanine core via a carbon‐carbon bond, whereas in compound **2**, a more flexible ether linkage is employed. Both have been recently investigated as potential photosensitizers in photodynamic therapy.^[^ ^37^ ^]^ The presence of charged substituents confers water solubility and prevents their aggregation via electrostatic repulsion at pH > 7,^[^ ^37^ ^]^ with the carboxylate functions predominantly oriented above and below the Pc ring. The absence of aggregation is evidenced by the sharp absorption bands centered at 700 and 680 nm, respectively for **1** and **2**, as well as by their strong fluorescence. The shift in absorbance to longer wavelengths, together with the slightly larger absorption extinction coefficient shown by **1** compared to **2** is likely a consequence of the direct conjugation between the isophthalate units and the Pc core. Both of the Pcs have also been shown to interact strongly with bovine serum albumin.^[^ ^37^ ^]^ Compound **3** is a commercially available tetra‐anionic derivative and was employed as a control for both the aggregation in aqueous media (broad absorbance with a maximum at 705 nm and very low absorption coefficient, Figure 1E; Figure S1A, Supporting Information, dashed green), as well as in the electrostatic self‐assembly (*vide* infra). The emission spectra of the dyes at 10 µm are presented in Figure 1F. The fluorescence maxima for **1** and **2** are located at 710 and 695 nm, respectively. As expected, dye **3** exhibits a heavily quenched fluorescence centered at 720 nm, due to its tendency for self‐aggregation (see Figure S1B, Supporting Information).

**Figure 2:**
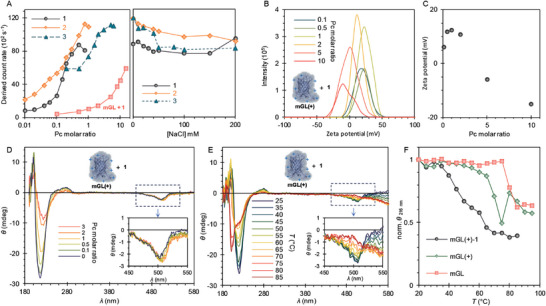
Structural characterization of protein‐dye complexes. A) Left: DLS of **mGL(+)** (0.5 mg mL^−1^) titrated with increasing dye concentrations. In addition, the **mGL** titrated with **1** is shown. Right: Protein‐dye complexes supplemented with increasing NaCl concentrations in DLS. B) ζ‐potential of **mGL(+)** (0.1 mg mL^−1^) with increasing **1** molar ratio. C) ζ‐potential maxima with the function of the **1** molar ratio. D) CD spectra of **mGL(+)** (0.5 mg mL^−1^) titrated with increasing **1** concentration. Inset: ellipticity between 450 and 550 nm. E) Temperature‐dependent CD spectra of **mGL(+)** (0.5 mg mL^−1^) with 1 molar equivalence of **1**. Inset: ellipticity between 450 and 550 nm. F) Ellipticity at 216 nm for **mGL(+)**, **mGL(+) **– **1** and **mGL**.

The protein‐dye complexation was initially studied with dynamic light scattering (DLS), monitoring the changes in scattering intensity (derived count rate) of the sample. **mGL(+)** (0.5 mg mL^−1^) was titrated with increasing Pc concentrations in buffered water (20 mm Tris, pH 8). After adding any of the three dyes, the scattering intensity rapidly increases, indicating a strong binding interaction between the protein and Pc, thus suggesting the formation of complexes (**Figure** **2**A, left). The maximum count rate is reached with ≈0.75 molar equivalents (13 µm) of Pcs **1** and **2**. Compound **3** reaches a maximum at 4 molar equivalents (88 µm), most likely due to the lower charge density of the compound. As a control, the native protein (**mGL**) was complexed together with **1**, and the results show a much smaller increase in the count rate, suggesting weaker binding and formation of smaller complexes. Nevertheless, determining the maximum binding equivalents in DLS is impeded at high ZnPc concentrations caused by the high ZnPc absorbance at the same wavelength as the measurement laser.

The nature of the binding was further studied by gradually adding NaCl to the preformed complexes. Typically, if the primary interaction between the two moieties is electrostatic, increasing the ionic strength of the solution induces charge screening, leading to complex disassembly.^[^ ^51^, ^52^, ^53^, ^54^, ^55^ ^]^However, the DLS results show almost no decrease in the scattering intensity upon increasing NaCl concentration (Figure 2A, right). This suggests that the interaction is remarkably strong due to the high charge density of both moieties.

Additionally, the complexation was studied by ζ‐potential measurements, where the **mGL(+)** (0.1 mg mL^−1^) was complexed with increasing molar ratios of **1** (Figure 2B,C). Adding low amounts of **1** to the **mGL(+)** forms positively charged complexes, with a maximum ζ‐potential value of 12.3 mV with 1 molar equivalences of **1**. As expected, further addition of **1** decreases the ζ‐potential as more negatively charged Pcs are adsorbed on the complex surface, shifting the slipping plane to reach negative values with 5 molar ratio of Pc.

To further study the interaction between the protein and the dyes, as well as the structural integrity of the protein, circular dichroism (CD) of the complex was measured (Figure 2D). Given the size and high charge density of the protein and dye molecules, it is expected that a strong interaction could lead to conformational distortions of the protein structure. Indeed, the electrostatic interaction between moieties causes a small (≈5 nm) shift in the ellipticity (*θ*) between 450 and 550 nm upon titration of **mGL(+)** (0.5 mg mL^−1^) with increasing concentration of **1** (Figure 2D, inset). This fact points toward some type of interaction between the protein chromophore and Pc, while preserving its chirality and, therefore, structural integrity.^[^ ^56^ ^]^ While more pronounced changes are visible in the far UV spectral fingerprint region of the barrel (216 nm), they are attributed to the formation of large complexes inducing scattering, rather than protein denaturalization. Additionally, titrating **mGL** with **1** (Figure S2A, Supporting Information) shows no decrease in *θ* or shift between 450 and 500 nm at similar dye concentrations as in **1**. Again, this implies that a cationic protein is required for efficient interaction with the anionic dye, reinforcing the hypothesis of electrostatic interactions as the main driving force in complex formations. The structural integrity was further characterized by temperature‐dependent CD. The **mGL(+)** remained intact up to 65 °C, after which the ellipticity at 216 nm started decreasing, suggesting a disintegration of the β‐barrel. The peak then shifts toward the far UV wavelengths at 80 °C but shifts back to 216 nm with higher temperatures, advocating the denaturation of the protein barrel (Figure S2B,F, Supporting Information). The residual signal is likely due to the presence of some remaining β‐sheets. A significant difference between the Pc titration and temperature degradation, however, is seen between 450 and 550 nm. The denaturation of the barrel causes the quenching of the chromophore signal (Figure S2B, Supporting Information, inset). As this is not observed in the titration experiments (Figure 2D, inset), these results further support that the addition of **1** does not interfere with the chromophore integrity. Nevertheless, temperature‐dependent measurements of the complex show that the complexation significantly decreases the stability of the protein (Figure 2E,F). Here, the ellipticity at 216 nm gradually increases from 40 °C upward and shifts toward the far UV region at 75 °C. These results suggest that the strong interaction might cause some conformational distortions to the protein structure without breaking the chromophore integrity but lowering the stability against thermal denaturation. As with **mGL(+)**, the ellipticity of the complex gradually increases around the chromophore region with the increasing temperature. As a control, **mGL** was measured with increasing temperatures and showed slightly higher stability against temperature denaturation compared to the supercharged protein. (Figure 2F; Figure S2C, Supporting Information). Modulated Scanning Fluorimetry (MSF)^[^ ^57^ ^]^ was used to characterize further the temperature denaturation of the **mGL(+)** and **mGL**, and the results are in line with CD (Figure S2G,H, Supporting Information). The data shows a gradual decrease in the protein fluorescence with the increase in temperature, fully quenching the fluorescence ≈85 °C for the **mGL(+)** and 92 °C for the **mGL**.

**Figure 3:**
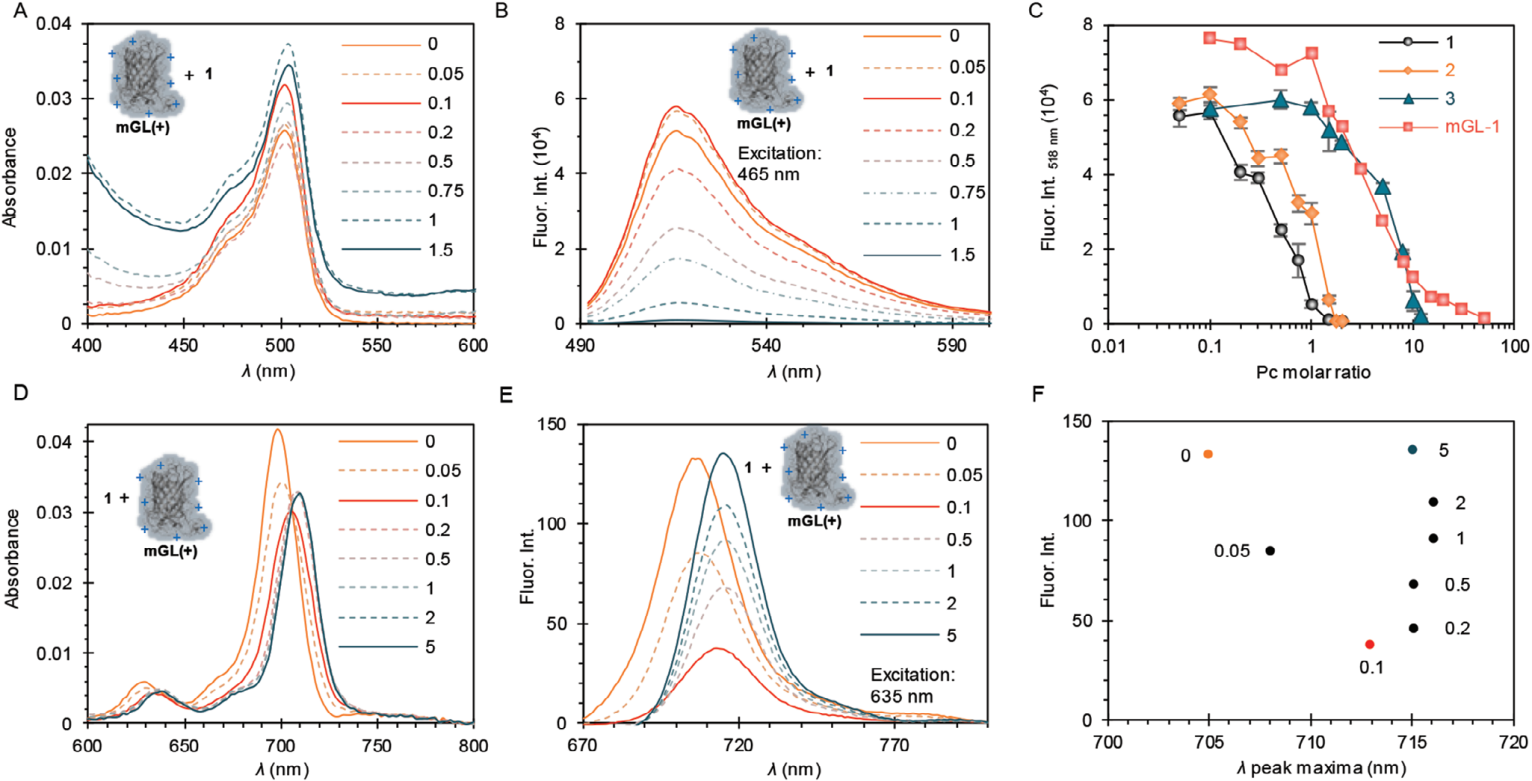
Photophysical characterization of the protein‐dye complex. A,B) **mGL(+)** (0.05 mg mL^−1^) supplemented with increasing concentrations of **1**. (A) Absorption spectra and (B) emission spectra (λ_ex_ = 465 nm). C) Fluorescence of **mGL(+)** at 518 nm with all dyes and **mGL** with **1** (λ_ex_ = 465 nm). The data shown is the average of three independent measurements with the standard deviation as error bars. D–F) Compound **1** (2 µM) titrated with increasing **mGL(+)** concentration. (D) Absorption spectrum. (E) Emission spectrum (λ_ex_  = 635 nm). F) The emission intensity against wavelength of emission maximum. The numbers indicate the molar equivalents of protein to **1**.

In order to unravel the photophysical characteristics between moieties, we studied the optical properties of the protein‐dye complex by means of absorption and fluorescence spectroscopy. The titration of **mGL(+)** (0.05 mg mL^−1^, 1.8 µm) upon increasing **1** concentration shows an increased scattering in the protein absorption spectrum, typical of the formation of large aggregates. However, no other noticeable changes were observed in the absorbance band centered at 480 nm (**Figure** **3**A; Figure S3A, Supporting Information). In the fluorescence spectra, the protein emission increases slightly in the presence of 0.1 molar equivalent of **1**, after which the intensity gradually decreases with the addition of dye until fully quenched at 1.5 molar equivalents (2.64 µm) (Figure 3B). This is in good agreement with the saturation point obtained by DLS. Similar behavior is detected upon titration with **2**, observing a fully quenched fluorescence at 2 molar equivalents (3.52 µm) (Figure 3C; Figure S4A,B, Supporting Information). As already suggested by DLS, Pc **3** shows similar behavior as **1** and **2**, but at higher equivalents (Figure S5A,B, Supporting Information). The protein fluorescence initially increases up to 0.5 molar equivalents of **3** (1.76 µm), until being fully quenched with 12 equivalents (21.1 µm). Moreover, the total fluorescence quenching suggests an interaction between the protein chromophore and the dye, in line with the CD results. Furthermore, as a control, **mGL(+)** was titrated with increasing molar equivalents of heparin (a negatively charged biopolymer), which had a minor effect on the protein fluorescence (Figure S6, Supporting Information). As a control, **mGL** (0.05 mg mL^−1^) was titrated with an increasing amount of **1** (Figure 3C; Figure S7, Supporting Information). The emission of the **mGL** is gradually decreased upon the addition of **1** and is fully quenched ≈50 molar equivalents (88.0 µm). These measurements confirm that the electrostatic interactions play a key role in the complex formation, and the Pcs interact with **mGL(+)** in the excited state.

The photophysical properties of the Pcs upon assembly were studied by titrating a constant concentration of **1** (2 µm) with increasing **mGL(+)** concentrations (Figure 3D–F). Both absorption and emission spectra show a slight red‐shift with increasing **mGL(+)** concentration. The overall shape of the absorption spectrum remains unchanged. However, the fluorescence of the Pc **1** is first decreased upon the addition of the protein, but then gradually regained at 5 molar equivalents (10 µm) of **mGL(+)** (Figure 3E–F). The redshift and changes in the fluorescence of Pc are likely the result of Pc ground‐state re‐arrangements upon hybrid formation.^[^ ^9^ ^]^ Pc **2** exhibits similar behavior, except the emission could not be fully recovered even with 5 molar equivalents of **mGL(+)** (Figure S4C,D, Supporting Information). Interestingly, titrating **3** (10 µm due to the lower absorption coefficient) with increasing molar concentrations of **mGL(+)** did not lead to a shift in either absorption or emission (Figure S5C,D, Supporting Information). However, the fluorescence measurements show quenching of the already weak Pc emission with 0.05 molar equivalents (0.5 µm) of **mGL(+)**, and then a recovery of the fluorescence with higher **mGL(+)** concentrations, even surpassing the initial fluorescence intensity. This is likely due to reducing its inherent self‐aggregation in water. Altogether, the fluorescence measurements are well in line with DLS results.

**Figure 4:**
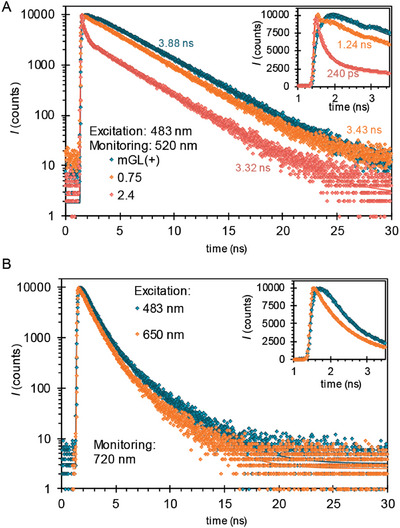
The emission decay profiles with TCSPC. A) Excitation of pure mGL(+) and mGL(+) – 1 complexes at 483 nm and monitoring the emission decays of mGL(+) at 520 nm. The inset shows the same decays presented in a narrower time interval. B) Excitation of 2.4 molar equivalent sample at 483 and 650 nm and monitoring the emission decays of Pc at 720 nm. The inset shows the same decay with a narrower time interval.

A more in‐depth photophysical characterization was carried out to further understand the photophysics in the **mGL(+)** – **1** complex. Thus, the fluorescence lifetimes were measured by emission decays of the photosystems with time‐correlated single photon counting (TCSPC). The complexes were excited at 483 nm and the emission time profile was monitored at 520 nm, i.e., at the **mGL(+)** fluorescence band. The emission decays for the reference **mGL(+)** (0.03 mg mL^−1^) and **mGL(+)** with 0.75 and 2.4 molar ratios of **1** (0.79 and 2.53 µm, respectively) are shown in **Figure** **4**. The decays were fitted to a bi‐exponential model, showing that a long‐lived component in all the decays, remains similar at approximately τ_2_ = 3 ns. However, a short‐lived component upon the addition of **1** can be observed, indicating that the addition of **1** results in a fast quenching of the **mGL(+)** fluorescence. The fast time constant decreases from 1.2 ns to 240 ps when the concentration of **1** is increased. To present the short‐lived component clearly, Figure 4A presents the emission decays in a narrower time interval with a linear intensity scale in the inset. The time constants are summarized in **Table** **1**.

**Table 1:** Fluorescence lifetimes of pure mGL(+) and mGL(+) – 1 complexes measured with 483 nm excitation.

Samples	τ_1_	τ_2_
**mGL(+)**	N.A.	3.88 ns
**mGL(+)** – **1** (0.75 molar eq of 1)	1.24 ns	3.43 ns
**mGL(+)** – **1** (2.4 molar eq of 1)	240 ps	3.32 ns

Since the lifetime of the longest component **mGL(+)** is in the same range for all the samples, the emission decays for the **mGL(+) – 1** complex at 2.4 equivalents of **1** were monitored at 720 nm, i.e., at the Pc fluorescence band, upon excitation at two wavelengths: at 483 nm, where **mGL(+)** shows dominant excitation, and at 650 nm to directly excite the Pc. The decays are shown in Figure 4B, where essentially no difference in the decay profiles at delay times longer than the instrument time resolution can be observed. However, in the early timescales, a fast‐rise component in the emission decay with 483 nm excitation can be observed and is attributed to a fast energy transfer process from **mGL(+)** to **1**. Unfortunately, quantitative analysis of this process cannot be performed due to the rise time being close to the time resolution limit of the instrument.

**Figure 5:**
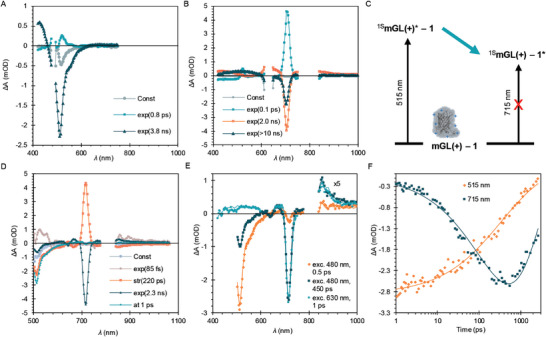
Transient absorption of the **mGL(+) **– **1** complex. A) Excitation at 480 nm to obtain TA decay‐associated spectra (DAS) of **mGL(+)**. B) Excitation at 630 nm to obtain TA decay associated spectra of **1**. C) Schematics of the photoinduced processes taking place in **mGL(+) **– **1** complex. D) DAS of **mGL(+) **– **1** excited at 480 nm. E) Time‐resolved TA spectra of **mGL(+) **– **1** at 0.5 and 450 ps delay and excited at 480 nm, and time‐resolved spectrum of the same sample but excited at 630 nm and taken at 1 ps delay time and scaled to match bleaching at 715 nm. F) TA decay profiles of **mGL(+) **– **1** at 515 and 715 nm were measured with excitation of 480 nm.

To further characterize the photophysics, the samples were investigated in the shorter timescale via employing ultra‐fast transient absorption (TA) spectroscopy. The samples were excited at two excitation wavelengths, 480 and 620 nm, and the transient absorption was monitored in both visible (420–750 nm) and near‐infrared (NIR) (850–1060 nm) ranges. First, pure **mGL(+)** (0.5 mg mL^−1^) was excited at 480 nm and its 2D TA map is shown in Figure S8 (Supporting Information). The sample shows photo‐induced absorbance in the 400–450 nm region and a negative ground state bleach is observed in the 450–500 nm range. The TA responses were fitted to a bi‐exponential model to obtain the decay‐associated spectra (DAS) and results are shown in **Figure** **5**A. The fast component with a time constant of 0.8 ps is relatively weak. This can be due to a nuclear rearrangement and is only a minor contribution. However, the long‐lived component with a time constant 3.8 ns is due to the excited state of **mGL(+)** (GLP*, Figure S8B, Supporting Information). Similarly, to analyze the photophysics of pure **1**, the sample was excited at 620 nm. Figure S9 (Supporting Information) shows the measured 2D TA map of **1** in which a strong ground state bleaching of the Q‐band can be observed at 700 nm. The data was fitted using a 3‐exponential model (Figure 5B) and the results were used to extract the singlet and triplet state spectra of **1** as shown in Figure S9B (Supporting Information). The lifetime of the singlet excited state is ≈2 ns, whereas the triplet state does not decay within our TA measurement scale (6 ns).

To investigate the energy transfer dynamics in the **mGL(+) – 1** complex at 2.4 molar equivalents (42.2 µm), the sample was first excited at 630 nm, as only **1** absorbs at this wavelength. The 2D TA map is shown in Figure S10A,B (Supporting Information). We initially fit the resulting decay using 3‐exponential global fitting. However, singular value decomposition (SVD) analysis^[^ ^59^ ^]^ of the data suggested that there is only one dominating component in the decay. Therefore, the data were fitted using a combination of a stretched exponential,^[^ ^60^ ^]^ a step function, and a constant (Figure S10C, Supporting Information). The constant is used to fit the probe signal nonuniformity, which does not depend on the delay time and is mostly caused by pump light scattering. The main component is the stretched exponential, which indicates a non‐exponential decay of the singlet excited state. The need to use a stretched exponential component is indicative of **1** having different transient absorption dynamics depending on the local environment and is most probably caused by the electrostatic interaction with **mGL(+)**. It has a time constant of 680 ps and a stretching factor of 0.54, which gives a formal average decay time constant of 930 ps.^[^ ^60^ ^]^ There is a small residual signal at long delay times given by the step function. It can be attributed to a small amount of triplet state formed after the relaxation of the singlet excited state. In general, this result resembles that obtained for pure **1**: the photoexcitation generates the **1** singlet excited state instantly, which decays to the triplet and ground states (Figure 5C). The main difference is a lower yield of the triplet state as the lifetime of the singlet excited state is reduced due to the distortions caused by the complex formation. This indicates that there is little to no electronic interaction between the excited state of **1** and **mGL(+)** in its ground state, and the only effect is conformational perturbations of **1** caused by the complex formation with **mGL(+)**.

Excitation of the complex at 480 nm changes the TA response drastically. The 2D TA maps are shown in Figure S10D,E (Supporting Information). SVD analysis suggests that two main intermediates in the excitation relaxation must be considered. The data were fitted using a stretched exponential and a long‐lived exponential component. In addition, a constant was added to account for the protein emission, and an additional fast exponential component was included to account for processes occurring at sub‐picosecond delay times. The results of the fit are presented in Figure 5D. The fast component (time constant 85 fs) can be attributed to thermal relaxation to the lowest singlet excited state along with possible instrumental artifacts. In contrast, the constant is needed to remove protein fluorescence and increase excitation light scattering. The main components are the stretched exponential (with a time constant 220 ps) and the long‐lived exponential (with a time constant 2.3 ns). A Comparison of the obtained time constants suggests that two characteristic delay times to distinguish between the intermediate states in the photophysical processes of **mGL(+) **– **1** are sub‐picoseconds after thermal relaxation and a few hundred picoseconds. The TA spectra of **mGL(+) **– **1** at 0.5 and 450 ps delay times after excitation at 480 nm are shown in Figure 5E, compared with a scaled TA spectrum of **mGL(+) **– **1** at 1 ps delay time after direct excitation of **1** at 630 nm. At short delay times, the TA spectrum of **mGL(+) **– **1** excited at 480 nm is almost identical to that of excited **mGL(+)** (Figure S8B, Supporting Information), except for a minor bleach at 715 nm, which is due to directly excited **1**. With time, the 515 nm bleach of **mGL(+)** decreases, and the 715 nm bleach of **1** increases. This process is described by the stretched exponential with a time constant 220 ps and stretching factor 0.54 (formal average time constant 380 ps), as illustrated in Figure 5F by the decay profiles at 515 nm (ground state bleaching of **mGL(+)**) and 715 nm (ground state bleaching of **1** Q‐band). This can also be seen in the DAS shown in Figure 5D, with the positive TA component of the stretched exponential indicating the formation of the bleach of **1** with the same time constant as the bleach of **mGL(+)** recovers. For comparison, the TA spectrum of **mGL(+) **– **1** at 1 ps delay time but after excitation of **1** at 630 nm is also shown in Figure 5E. The spectra at 450 ps after excitation of **mGL(+)** at 480 nm and at 1 ps after direct excitation of **1** are almost identical at wavelengths above 600 nm. These results suggest an energy transfer process occurring from **mGL(+)** to **1**. The average time constant of energy transfer obtained from TAS measurements agrees with the time constant obtained from the TCSPC emission decay measurements considering the large differences in time resolution (sub ps vs sub ns) and measurement time scales (a few ns vs hundreds of ns).

It is noteworthy that in the **mGL(+) **– **1** complex used for the TA study, the energy transfer is only a few times faster than the lifetimes of the singlet excited states of both **mGL(+)** and **1** (in **mGL(+) **– **1** complex) as can be seen from Figure S10F (Supporting Information). This complicates the quantitative analysis of the energy transfer process. Another complication in the analysis is a lack of knowledge of the exact kinetics of the reaction. It is reasonable to expect some distribution of distances and mutual orientation of transition dipole moments of the energy donor and acceptor, but the used stretched exponential approximation is an empirical model, which cannot provide any information on the affected physical parameters. However, it allows us to estimate an average time scale of the process. Due to the lack of spectral overlap between the protein and the Pc, the energy transfer process is tentatively anticipated to be a Dexter type. The slight distortion in the protein β‐barrel might allow a partial insertion of the Pc into the barrel, bringing it to the proximity of the chromophore and facilitating the wavefunction overlap between the Pc and the protein chromophore. However, further studies to determine the exact energy transfer mechanism are needed.

## 3 Conclusion

Bio‐based materials, especially fluorescent proteins, are promising candidates for photoactive materials in water‐based photosynthetic systems. Here, we studied the structural and photophysical properties of self‐assembled protein‐dye complexes. A positively supercharged mGreenLantern protein was complexed with highly anionic, nonaggregating phthalocyanine molecules. The results show that protein and Pc have a strong interaction, which is mainly driven by electrostatic interactions. The results suggest that the Pc causes slight conformational changes to the protein barrel, however, without compromising the integrity of the chromophore. The photophysical characterization of the system shows quenching of the **mGL(+)** fluorescence upon the addition of Pc. Studying the fluorescence lifetime of the complex revealed that the addition of Pc into the system results in a fast quenching of the protein excited state emission, which can be associated with fast energy transfer. The photophysical characterization of the complex with TA spectroscopy suggests energy transfer from the protein to phthalocyanine upon exclusive excitation of the protein with time constants that are consistent with the ones obtained from TCSPC measurements. We anticipate that our highly modular approach of supramolecular self‐assembled bio‐inspired materials can act as a starting point for developing “mix‐and‐match” photoactive materials with rich photophysical properties.

## 4 Experimental Section

### Materials

**mGL(+)** prepared as described in previously reported methods.^[^ ^20^ ^]^ **mGL** was expressed and purified following the same procedure. **1** and **2** were prepared as described previously.^[^ ^37^ ^]^ Zinc(II) 2,9,16,23‐tetra(carboxy)phthalocyanine (**3**) was purchased from Porphychem (catalog no. 160).

### Buffer Exchange

The buffer for **mGL(+)** was changed from the 20 mm Tris, 500 mm NaCl storage buffer to 20 mm Tris, pH 8. A 100 µL protein stock was thawed from −80 °C and centrifuged in a tabletop centrifuge for 30 min. The supernatant was removed and dialyzed overnight at +4 °C using 3.5 kDa MWCO Slize‐A‐Lyzer Mini Dialysis cups (ThermoScientific). The concentration of the supernatant was then determined by UV–vis. ε_mGL(+)_ = 90 650 m ^−1^ cm^−1^.

### Electrostatic Potential

The electrostatic potential was visualized using PDB2QPR and Adaptive Poisson‐Bolzmann Solver (APBS) tools.^[^ ^61^ ^]^ PDB2PQR was first used to predict the protonation states, in which the pH was set to 8, PROPKA was used to assign protonation states and the force field was set as parse. Then, the APBS was used to compute the electrostatic potential with mg‐auto calculation type. The electrostatic potential was visualized with Chimera V. 1.17.3 between −10 k_B_ and 10 k_B_T e^−1^.

### Dynamic Light Scattering

The count rate of the complexes was measured using a Malvern Instruments DLS device (Zetasizer Nano ZS Series) with a 4 mW He‐Ne gas laser at a wavelength of 633 nm and a detection angle of 173°. All the measurements were done at room temperature. Disposable 1.5 mL PMMA cuvettes (Brand) were used. Zetasizer software (Malvern Instruments) was used to obtain the count rate. 0.5 mg mL^−1^ of **mGLP(+)** or **mGL** in 20 mm Tris, pH 8 was titrated with different Pc concentrations. No dilution correction was done as the total addition did not exceed 10% sample volume. After reaching the desired ratio, the samples were titrated with 5 m NaCl to disassemble the complex.

### ζ‐Potential

The ζ‐potential of the complexes was measured using a Malvern Zetasizer Nano ZS90 with 90° optics and a 633 nm laser. The measurements were done using Malvern Panalytical ZEN1002 dip cell. Each sample contained 0.1 mg mL^−1^ of **mGL(+)** in 20 mm Tris, pH 8, supplemented with 10 mM NaCl. Zetasizer software (Malvern Instruments) was used to obtain the ζ‐potential.

### Circular Dichroism

The circular dichroism with constant protein concentration (0.5 mg mL^−1^) and increasing Pc concentrations were measured in 1 mm path‐length quarts cuvettes (Hellma) with Jasco J‐1500 instrument. Ten accumulated scans for each sample with baseline correction were measured between 750 and 190 nm, with 1 nm resolution at room temperature. For the temperature‐dependent measurements, five accumulated scans for every 5 °C (20–95 °C) were measured between 780 and 180 nm with baseline correction.

### Modulated Scanning Fluorimetry

The Thermocycler CFX96 Touch Real‐time PCR System (Bio‐Rad) was employed with the standard program composed of heating and cooling cycles ranging from 25 to 99 °C to measure the progressive loss of fluorescence and the irreversible unfolding of the protein. The samples were heated at 5 °C s^−1^ and held for 1 min at the temperature peak, followed by a recovery period of 5 min at 25 °C. The proteins (1 µm) were added per well to avoid saturation. The thermograms were buffer‐subtracted and normalized by the highest fluorescence read‐out of each sample. Data analysis was performed using Origin 2019 (OriginLab Corporation, Northampton, MA, USA). Mean values and standard deviations of quintuplicate were calculated and plotted. Melting curves were obtained by plotting the fluorescence values obtained at peak temperatures, while nonreversibility curves were obtained by plotting the fluorescence values obtained at 25 °C.

### Photophysical Characterization

UV–vis and fluorescence spectra were measured using a Cytation 3 plate reader (BioTek) in 96‐well plates. Absorption spectra were measured using a wavelength range 400–800 nm. The fluorescence spectra for the protein titrations (0.05 mg mL^−1^) were measured with an excitation wavelength of 465 nm, and a range of 492–700 nm with a gain of 85. Prior to time‐resolved fluorescence and transient absorption measurements, the steady‐state fluorescence spectra were recorded by employing Fluorolog Yobin Yvon‐SPEX fluorometer. The excitation wavelength was 483 nm, and the spectra were automatically corrected using a correction function provided by the manufacturer.

### Fluorescence Spectrofluorometry

Emission spectrums for the Pc were measured using a Cary Eclipse spectrofluorometer (Agilent). For **1**, the fluorescence was measured between 650 and 800 nm with an excitation wavelength of 635 nm. Both, excitation and emission slits were set to 10, scan rate was 12 nm min^−1^ and the PMT voltage was medium. For **2**, the fluorescence was measured between 640 and 800 nm with an excitation wavelength of 620 nm. Again, both slits were set to 10, scan rate was 12 nm min^−1^ and the PMT voltage was medium. For **3**, the fluorescence was measured between 665 and 800 nm with an excitation wavelength of 635 nm. The slits were set to 20, scan rate was 120 nm min^−1^, and the PMT voltage was high.

### Time‐Resolved Fluorescence

**mGL(+)** (0.03 mg mL^−1^) and 0.75 and 2.4 molar equivalents of **1 (**0.79 and 2.53 µm, respectively). The time‐resolved fluorescence was measured using a time‐correlated single photon counting (TCSPC) system (Pico‐Quant GmBH) consisting of a PicoHarp 300 controller and a PDL 800‐B driver. Three excitation sources (pulsed laser diode) were used, emitting at 375, 483, and 650 nm. The time resolutions are slightly different with different diodes, been roughly (FWHM) 80, 120, and 90 ps, respectively. The signals were detected with a microchannel plate photomultiplier tube (Hamamatsu R2809U). The influence of the scattered excitation light was reduced with a cutoff filter in front of the monitoring monochromator. Fluorescence decays were collected with a constant accumulation time in the 500–630 nm wavelength range with steps of 10 nm. The instrumental response function (IRF) was measured separately, and the decays were simultaneously deconvoluted and fitted by applying the iterative least‐squares method either to the sum of exponents by using Equation (1):

(1) $$I\left(t,\lambda \right)=\sum_{i}a_{i}\left(\lambda \right)e^{-t/\tau_{i}}$$

### Transient Absorption Spectroscopy

**mGL(+)** (0.5 mg mL^−1^) complexed with **1** with 2.4 molar equivalents (42.2 µm). For transient absorption spectroscopy measurements, ultrafast time‐resolved pump‐probe spectroscopy was employed. The fundamental laser pulses were generated via Ti:Sapphire laser (Libra F, Coherent Inc., center wavelength of 800 nm, ≈100 fs pulse width, repetition rate 1 kHz). Almost 90% of the fundamental beam was sent to the optical parametric amplifier (OPA; Topas C, Light Conversion Ltd.) to generate tunable pump pulses to excite the samples at the desired wavelength. The remaining 10% of the fundamental beam was delivered to the motorized stage (delay line) and then to a 2 mm cuvette with water to generate continuum white light for probe pulses. The probe light was split into the reference and signal beams. The absorbance change was measured in chopper mode which is synchronized with fundamental laser pulses. The samples were excited at 480 and 620 nm, and the spectra were averaged over 3000 excitation pulses for each delay time. The spectra were taken out in both visible (500–775 nm) and NIR (840–1060 nm) ranges. The TA data were fitted globally by utilizing either exponential (Equation 1) or a sum of exponent and stretched exponential decay function to obtain decay‐associated spectra (DAS) and corresponding time constants. In the latter case, the carrier recombination can be presented by a stretched exponential^[^ ^62^ ^]^ as shown in **Equation** (**2**), where τ_𝑠𝑡𝑟_ is the stretched time constant and 𝛽 (0 < 𝛽 ≤ 1) is the stretching parameter.

(2) $$\Delta A\;\;\left(\lambda ,t\right)=a_{0}\;exp^{-{\left(\frac{t}{\tau_{str}}\right)}^{\beta }}$$

### Statistical Analysis

All the absorption spectra were buffer‐subtracted. The absorption and emission spectra in Figure 1C have been normalized by the highest read‐out of each measurement. The absorption and emission spectra are presented as the average of three independent measurements (Figure 2B,C; Figures S3–S5, Supporting Information). The fluorescence endpoints are presented as the average of three independent measurements with standard deviation as the error bars (Figure 3C). The temperature‐dependent CD measurements are smoothened by averaging three consecutive data points (Figure 2E; Figure S2B,C, Supporting Information). The data in 2F has been normalized by the read‐out at 20 °C. MFS data is presented as the mean values of quintuplicate measurements with the standard deviation as the error. The thermograms were buffer‐subtracted and normalized by the highest fluorescence read‐out of each sample.

## Conflict of Interest

The authors declare no conflict of interest.

## Supporting information



Supporting information

## Data Availability

The data that support the findings of this study are available from the corresponding author upon reasonable request.
