# Supplementary material for: Elucidating the Supramolecular Interaction of Positively Supercharged Fluorescent Protein with Anionic Phthalocyanines
Source: Adv Biol (Weinh). 2024 Oct 16;9(5):2400308. doi: 10.1002/adbi.202400308 (PMC12078875; doi:10.1002/adbi.202400308)
Supplement: Supplementary file 1 — Supporting information [file ADBI-9-2400308-s001.pdf]

ADVANCED BIOLOGY

Supporting Information

for *Adv. Biology*, DOI 10.1002/adbi.202400308

Elucidating the Supramolecular Interaction of Positively Supercharged Fluorescent Protein with Anionic Phthalocyanines

Sharon Saarinen, Ramsha Khan, Marta Patrian, Juan Pablo Fuenzalida-Werner, Rubén D. Costa, Petr Zimcik, Veronika Novakova, Tero-Petri Ruoko, Nikolai V. Tkachenko, Eduardo Anaya-Plaza and Mauri A. Kostiainen**

Supporting Information

Elucidating the supramolecular interaction of positively supercharged fluorescent protein with anionic phthalocyanines

Sharon Saarinen, Ramsha Khan, Marta Patrian, Juan Pablo Fuenzalida-Werner, Rubén D. Costa, Petr Zimcik, Veronika Novakova, Tero-Petri Ruoko, Nikolai V. Tkachenko, Eduardo Anaya-Plaza, and Mauri A. Kostiainen**

Contents

Note S1: Absorption and emission spectra for **3** S3
Note S2: CD spectra and MFS S4
Note S3: Full absorption spectra for **1** S5
Note S4: Photophysical properties with **2** S6
Note S5: Photophysical properties with **3** S7
Note S6: Heparin titration........ S8
Note S7: mGL titration with **1** S9
Note S8: Transient absorption of **mGL(+)** S10
Note S9: Transient absorption of compound **1** S11
Note S10: Transient absorption of **mGL(+) – 1** complexes........ S12

Note S1: Absorption and emission spectra for 3

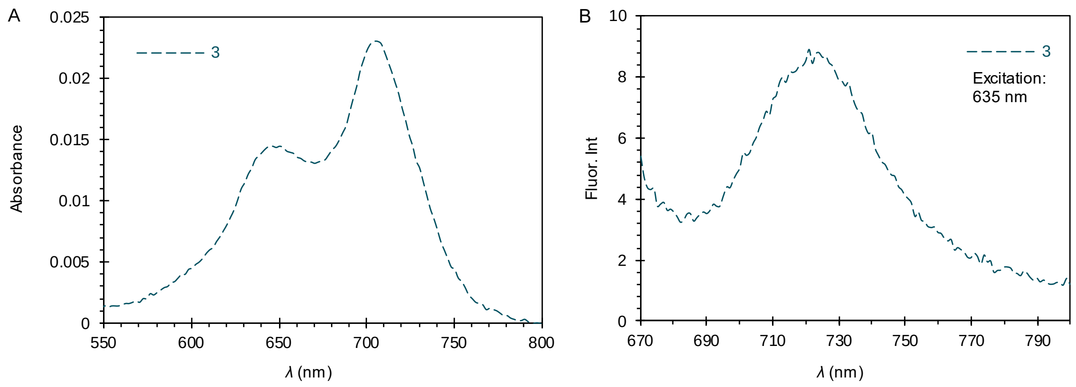

Figure S1. The absorption and the emission spectra for **3**. (A) The absorption spectrum at 10 µM concentration. (B) Emission spectrum measured with 100 µM concentration, an excitation of 635 nm and both excitation and emission slits set to 20 due to the low absorption coefficient of the dye.

Note S2: CD spectra and MFS

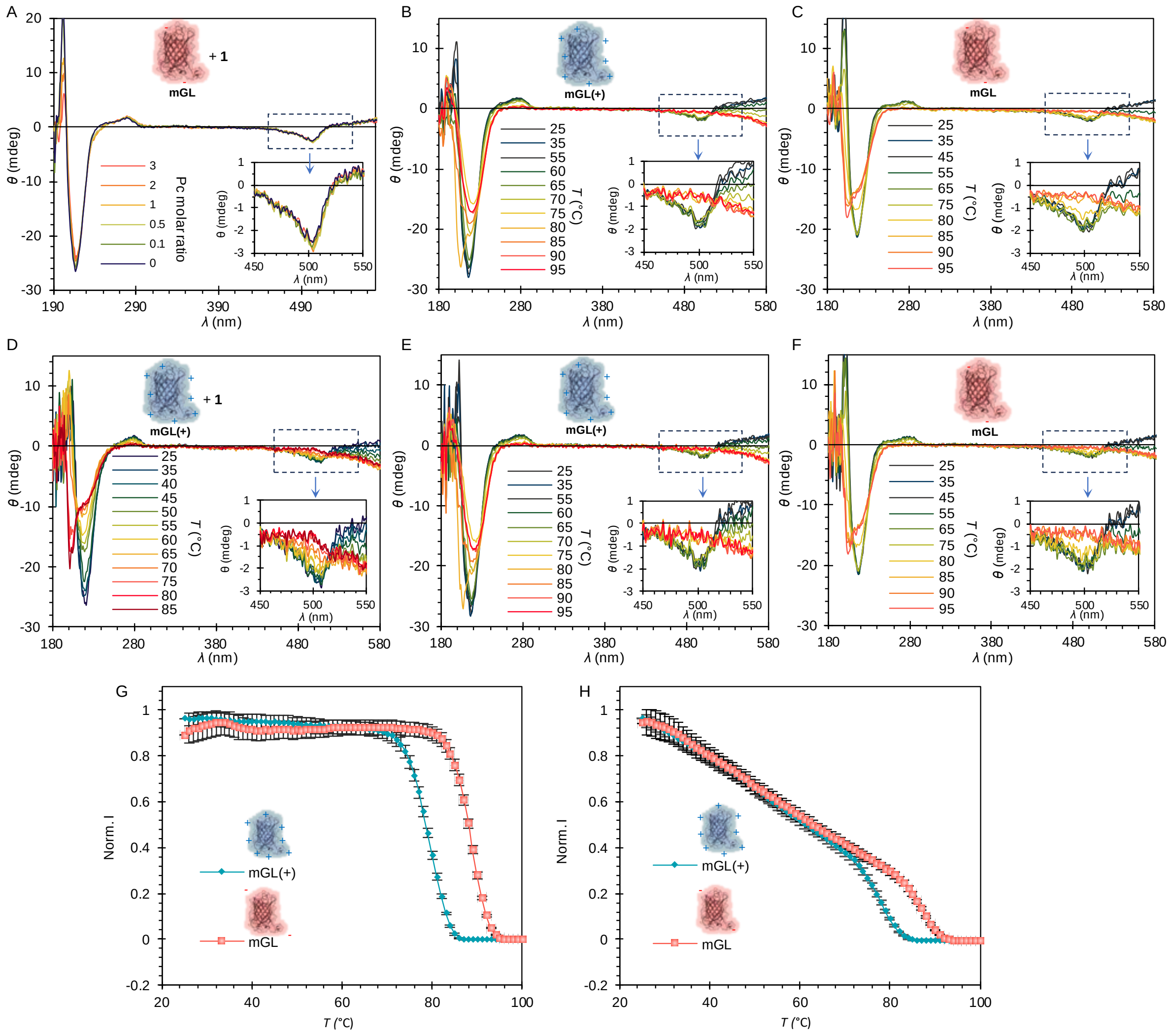

Figure S2. The CD spectra and MSF. (A) The full CD spectrum for titration of **mGL** (0.5 mg/ml) with increasing **1** concentration. Pc molar ratio indicates the molar equivalents of **1** to the protein. (B) Temperature-dependent CD of **mGL(+)**. Inset: ellipticity between 450 and 550 nm. (C) Temperature-dependent CD of **mGL**. Inset: ellipticity between 450 and 550 nm. (B–C) The data has been smoothened by averaging three consecutive data points. (D–F) Original temperature-dependent CD data for (D) **mGL(+)** with 1 molar equivalence of **1**, (E) **mGL(+)** and (F) **mGL**. (G) Temperature of non-reversibility measured in MSF. (H) MSF with increasing temperatures.

Note S3: Full absorption spectra for 1

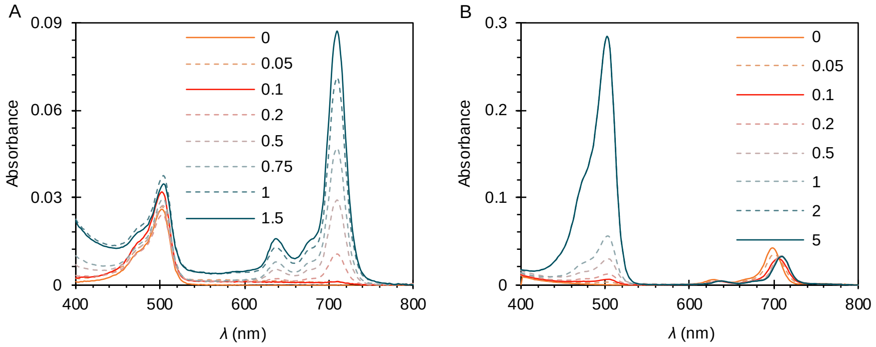

Figure S3. The full absorption spectra for (A) titrating **mGL(+)** (0.05 mg/ml) with increasing **1** molar equivalents and (B) titrating **1** (2 µM) with increasing **mGL(+)** concentrations.

Note S4: Photophysical properties with 2

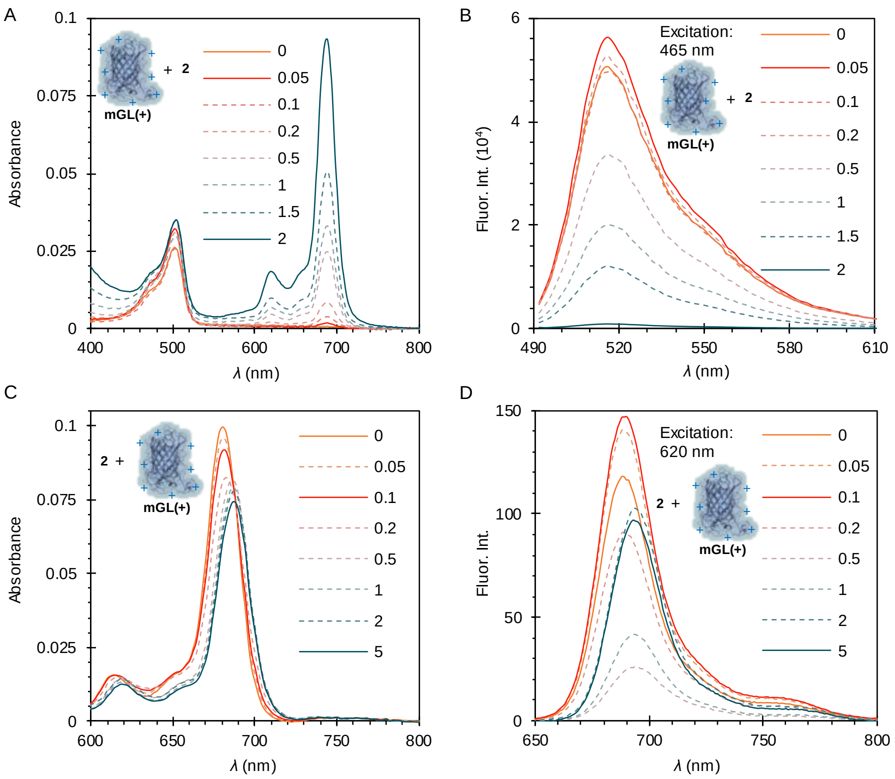

Figure S4. The photophysical properties of **mGL(+)** and **2**. (A) Absorption and (B) emission spectra of **mGL(+)** (0.05 mg/ml) titrated with increasing molar equivalents of **2**. Excitation was 465 nm. (C) Absorption and (D) emission spectra for constant dye (2 µM) with increasing **mGL(+)** concentrations. The sample was excited at 620 nm and both emission and excitation slits were set to 10.

Note S5: Photophysical properties with 3

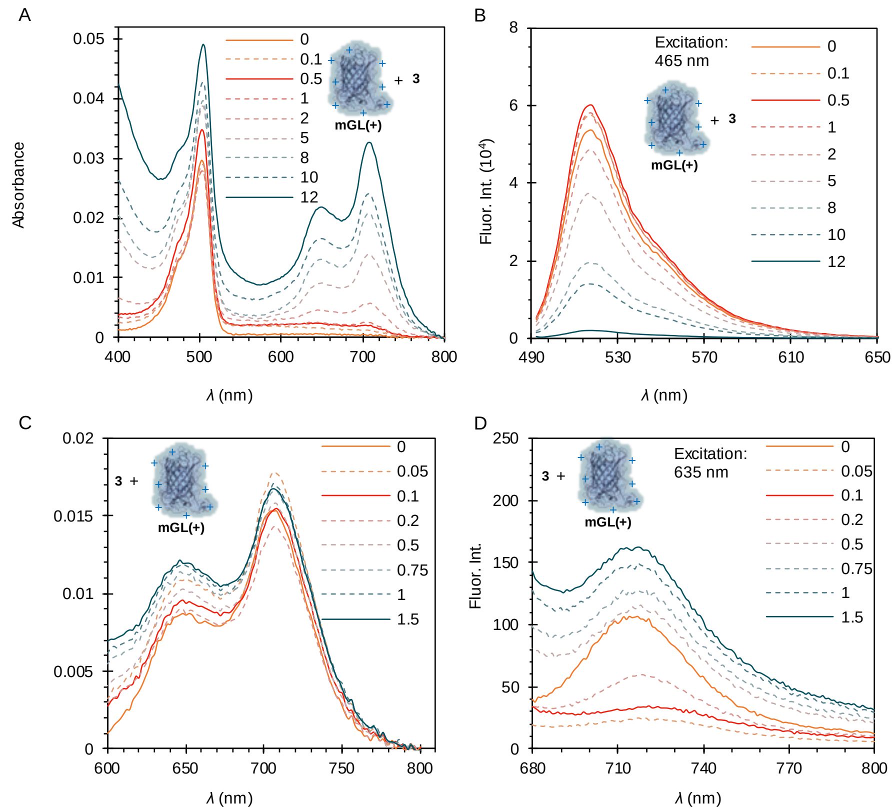

Figure S5. The photophysical properties of **mGL(+)** and **3**. (A) Absorption and (B) emission spectra of **mGL(+)** (0.05 mg/ml) titrated with increasing molar equivalents of **3**. Excitation was 465 nm. (C) Absorption and (D) emission spectra for constant dye (10 µM) with increasing **mGL(+)** concentrations. The sample was excited at 635 nm and both excitation and emission slits were set to 20, due to the lower absorption coefficient of the dye.

Note S6: Heparin titration

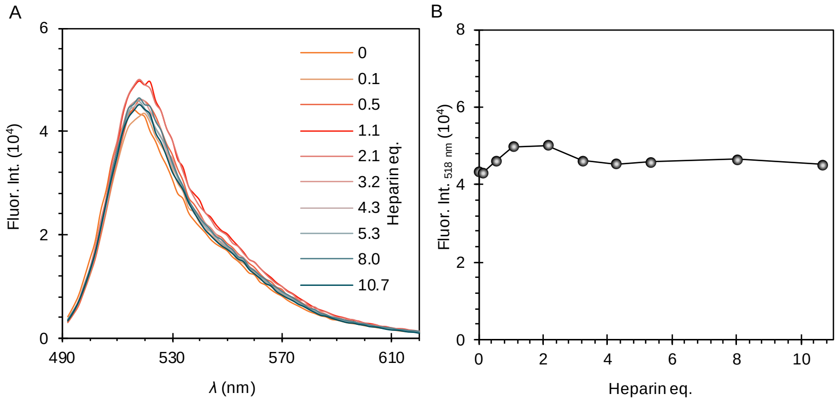

Figure S6. Titrating the **mGL(+)** (0.05 mg/ml) with increasing molar equivalents of heparin. (A) Emission spectra with an excitation of 465 nm. (B) Emission at 518 nm.

Note S7: mGL titration with 1

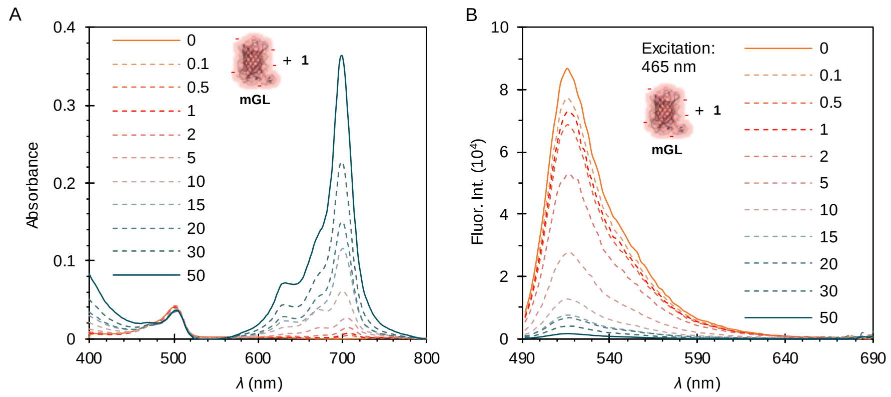

Figure S7. Titrating **mGL** (0.05 mg/ml) with an increasing **1** molar equivalents as a control. (A) Absorption and (B) emission spectra. Emission measured with an excitation of 465 nm.

Note S8: Transient absorption of mGL(+)

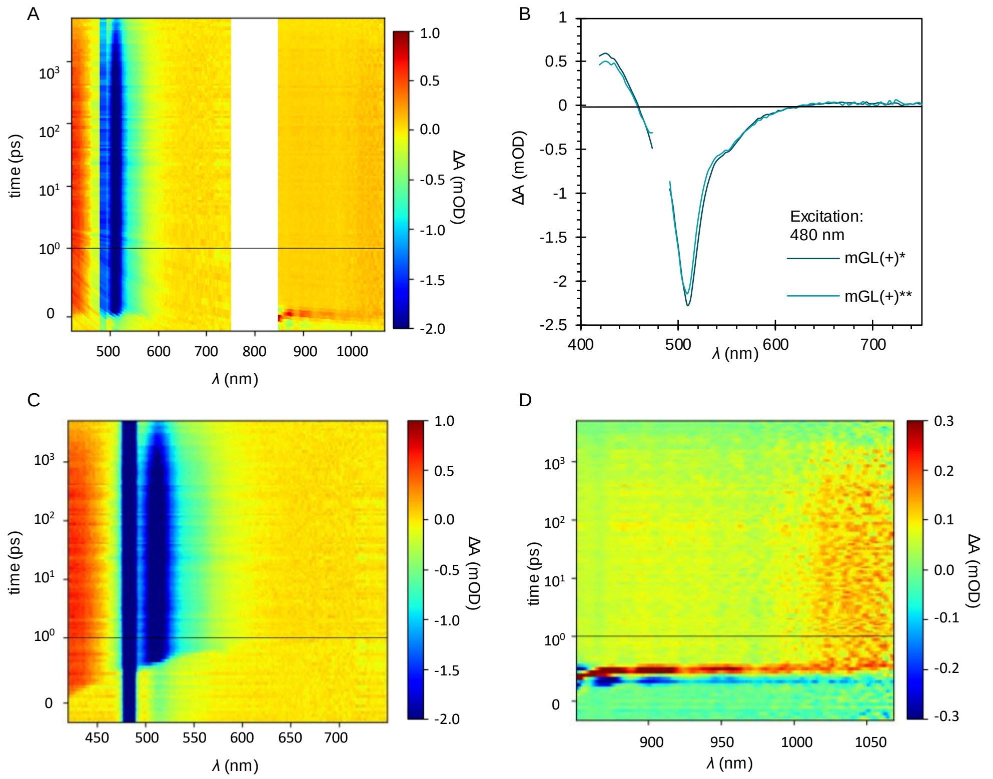

Figure S8. Transient absorption of **mGL(+)** (A) 2D TA spectra of **mGL(+)** (λ_{ex} = 480 nm). (B) TA spectra of **mGL(+)** excited states before and after fast (0.8 ps) relaxation, **mGL(+)**** and **mGL(+)***, respectively. (C—D) Non-corrected 2D TA spectra in (C) visible and (D) NIR ranges for **mGL(+)**.

Note S9: Transient absorption of compound 1

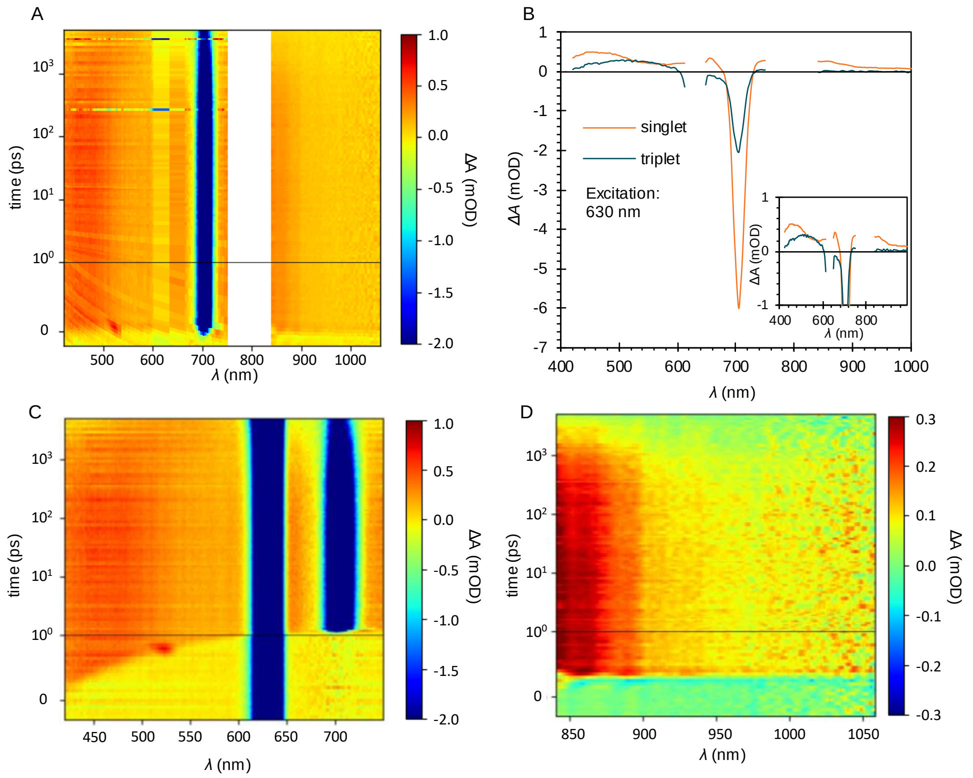

Figure S9. Transient absorption of **1.** (A) 2D TA spectra of **1** (λ_{ex} = 630 nm). (B) **1** singlet and triplet states obtained from TA measurements. (C—D) Non-corrected 2D TA spectra in (C) visible and (D) NIR ranges for **1**.

Note S10: Transient absorption of mGL(+) – 1 complexes

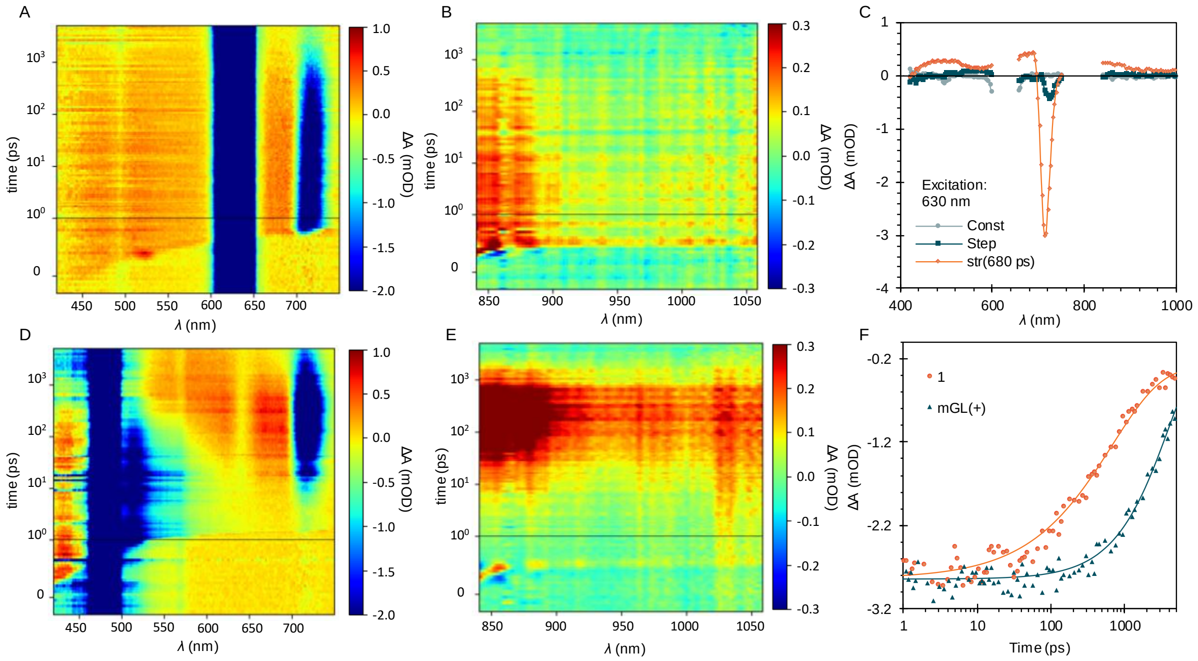

Figure S10. Transient absorption of **mGL(+) – 1** complex**.** (A—B) 2D TA spectra of **mGL(+) – 1** in (A) visible and (B) NIR ranges excited at 630 nm. (C) The associated DAS of **mGL(+) – 1** excited at 630 nm. (D—E) 2D TA spectra in (D) visible and (E) NIR ranges for **mGL(+) – 1** complex excited at 480 nm (F) TA decay profiles of **mGL(+) – 1** decay of at 715 nm but excited at 630 nm (**1**), and decay of **mGL(+)** at 515 nm excited at 480 nm (**mGL(+)**); **1** and **mGL(+)** traces were scaled for easier decay comparison.
